# Traffic-related air pollution and supplemental folic acid intake in relation to DNA methylation in granulosa cells

**DOI:** 10.1186/s13148-023-01503-y

**Published:** 2023-05-13

**Authors:** Audrey J. Gaskins, Robert B. Hood, Jennifer B. Ford, Russ Hauser, Anna K. Knight, Alicia K. Smith, Todd M. Everson

**Affiliations:** 1 Department of Epidemiology, Rollins School of Public Health, Emory University, 1518 Clifton Road, Atlanta, GA 30322 USA; 2 Department of Environmental Health, Harvard T.H. Chan School of Public Health, Boston, MA 02115 USA; 3 Department of Gynecology and Obstetrics, School of Medicine, Emory University, Atlanta, GA 30322 USA; 4 Gangarosa Department of Environmental Health, Rollins School of Public Health, Emory University, Atlanta, GA 30322 USA

**Keywords:** Air pollution, Epigenetics, Fertility, Folate, Ovary, Granulosa

## Abstract

**Background:**

Higher exposure to traffic-related air pollution (TRAP) is related to lower fertility, with specific adverse effects on the ovary. Folic acid may attenuate these effects. Our goal was to explore the relation of TRAP exposure and supplemental folic acid intake with epigenetic aging and CpG-specific DNA methylation (DNAm) in granulosa cells (GC). Our study included 61 women undergoing ovarian stimulation at a fertility center (2005–2015). DNAm levels were profiled in GC using the Infinium MethylationEPIC BeadChip. TRAP was defined using a spatiotemporal model to estimate residence-based nitrogen dioxide (NO_2_) exposure. Supplemental folic acid intake was measured with a validated food frequency questionnaire. We used linear regression to evaluate whether NO_2_ or supplemental folic acid was associated with epigenetic age acceleration according to the Pan-tissue, mural GC, and GrimAge clocks or DNAm across the genome adjusting for potential confounders and accounting for multiple testing with a false discovery rate < 0.1.

**Results:**

There were no associations between NO_2_ or supplemental folic acid intake and epigenetic age acceleration of GC. NO_2_ and supplemental folic acid were associated with 9 and 11 differentially methylated CpG sites. Among these CpGs, only cg07287107 exhibited a significant interaction (*p*-value = 0.037). In women with low supplemental folic acid, high NO_2_ exposure was associated with 1.7% higher DNAm. There was no association between NO_2_ and DNAm in women with high supplemental folic acid. The genes annotated to the top 250 NO_2_-associated CpGs were enriched for carbohydrate and protein metabolism, postsynaptic potential and dendrite development, and membrane components and exocytosis. The genes annotated to the top 250 supplemental folic acid-associated CpGs were enriched for estrous cycle, learning, cognition, synaptic organization and transmission, and size and composition of neuronal cell bodies.

**Conclusions:**

We found no associations between NO_2_, supplemental folic acid, and DNAm age acceleration of GC. However, there were 20 differentially methylated CpGs and multiple enriched GO terms associated with both exposures suggesting that differences in GC DNAm could be a plausible mechanism underlying the effects of TRAP and supplemental folic acid on ovarian function.

**Supplementary Information:**

The online version contains supplementary material available at 10.1186/s13148-023-01503-y.

## Introduction

Air pollution, particularly due to motor vehicles, is a ubiquitous exposure and significant global health threat [1]. Emerging evidence suggests that traffic-related air pollution (TRAP) is related to lower human fertility [2], with specific adverse effects on female reproductive function including impaired folliculogenesis [3–5]. On the other hand, preconception folic acid intake, particularly at doses higher than those recommended for the prevention of neural tube defects, has been consistently related to improved reproductive success [6–9]. High folic acid intake has also been shown to attenuate the adverse effects of air pollution on congenital heart defects, autism, and fertility [10–12], yet the underlying mechanism remains unclear. Since both folic acid and TRAP appear to have specific effects on the ovary, a large part of their observed associations with fertility are likely mediated through effects on the female gametes.

Given that TRAP has been related to hypomethylation in peripheral blood leukocytes and the placenta [13–15] and dietary folate is a key source of methyl-groups [16], epigenetics, and DNA methylation (DNAm) specifically, is a predominant biological pathway of interest. An intervention study showed that a B-vitamin supplement containing a high dose of folic acid could directly prevent the global hypomethylation of DNA in blood leukocytes following TRAP exposure [17]. However, tissue specificity renders it difficult to extrapolate global and gene-specific DNAm findings from circulating cells to reproductive tissues such as the ovaries [18]. Additionally, TRAP and folic acid exposure have been related to epigenetic aging metrics [19, 20], which are calculated using DNAm values from age-associated CpG sites. This has led to the hypothesis that TRAP accelerates epigenetic aging while folic acid (and other B-vitamins) may slow it. Since the ovaries are one of the fastest aging organs and chronological age is the strongest predictor of female fertility [21], any exposures that affect the epigenetic age of the ovary will undoubtedly have important consequences on reproductive function [22]. Recent work shows that granulosa cells (GCs) have the largest error in predicted age compared to other tissues when age is predicted by the multi-tissue DNAm clock, indicating that ovarian somatic cells age epigenetically differently and independently than other tissue types [23, 24]. However, no studies have yet examined whether TRAP and folate impact epigenetic aging or loci-specific DNAm specifically in ovarian somatic cells.

Our primary hypothesis is that TRAP exposure during folliculogenesis accelerates epigenetic aging, and possibly modulates epigenetic regulation of GCs, which may affect oocyte development, and that high intake of supplemental folic acid may counteract this. Thus, our objective was twofold: (1) to evaluate whether there are differences in DNAm age acceleration in GCs among women with high vs. low TRAP exposure and supplemental folic acid intake and (2) to explore whether there are differentially methylated CpG sites among women with high vs. low TRAP exposure and supplemental folic acid intake.

## Results

### Study flow and characteristics

Of the 132 women who had a stored GC aliquot sent for analysis, one sample was broken in transit. DNA extraction yields from the remaining 131 samples were variable, ranging from 0 to 2896 ng. Only 45% (n = 60) had ≥ 200 ng and 12% (n = 16) had 100–199 ng of DNA extracted. There were no associations between the exposures (or any of the other covariates) and DNA yield. We included 64 samples on the DNAm array, including four with 100–199 ng of DNA. Following quality control and processing of the methylation data, we excluded two samples where > 5% of probes yielded detection *p*-values > 0.001 and one sample that was an outlier in principal components analyses, leaving a final sample size of 61 women.

The 61 women included in our final analysis had a median (interquartile range, IQR) age of 34.8 (5.7) yrs and body mass index (BMI) of 24.2 (5.5) kg/m^2^. The majority were White (87%), non-Hispanic (93%), never smokers (74%), with a college degree or higher (92%) (Table 1). Primary infertility diagnoses at enrollment included male factor (28%), female factor (36%), and unexplained (36%) causes. Women who were categorized as having high (> 34.1 ppb NO_2_ exposure) vs. low (< 11.7 ppb NO_2_ exposure) TRAP exposure had a median (IQR) nitrogen dioxide (NO_2_) exposure of 43.3 (27.7) and 9.2 (5.1) ppb during the three months prior to controlled ovarian stimulation, respectively. Similarly, women with high (≥ 800 µg/day) vs. low (< 400 µg/day) supplemental folic acid intake had a median (IQR) intake of 829.8 (200.0) and 230.6 (342.9) µg/day, respectively.

**Table 1:** Demographic and reproductive characteristics of the women selected for and included in our pilot study on TRAP exposure, supplemental folic acid intake, and granulosa cell DNA methylation

Median ± IQR or N (%)	Women with GC samples sent for analysis (n = 132)	Women included in the GC DNAm analysis (n = 61)
*Demographic characteristics*		
Age, years	34.8 ± 5.8	34.8 ± 5.7
BMI, kg/m^2^	23.1 ± 5.0	24.2 ± 5.5
Smoking status		
Never	96 (72.7)	45 (73.8)
Past/Current	36 (27.3)	16 (26.2)
Race		
White	112 (84.9)	53 (86.9)
Black	2 (1.5)	0 (0.0)
Asian	14 (10.6)	5 (8.2)
Other	4 (3.0)	3 (4.9)
Ethnicity		
Hispanic	10 (7.6)	4 (6.6)
Non-hispanic	122 (92.4)	57 (93.4)
Education		
High school graduate	4 (3.0)	2 (3.3)
Some college	7 (5.3)	3 (4.9)
College graduate	45 (34.1)	23 (37.7)
Graduate degree	76 (57.6)	33 (54.1)
NO_2_ Exposure, ppb		
High	42.0 ± 16.5	43.3 ± 27.7
Low	9.7 ± 6.4	9.2 ± 5.1
Supplemental folic acid intake, µg/day		
High	800.2 ± 200.0	829.8 ± 200.0
Low	228.6 ± 342.9	230.6 ± 342.9
*Reproductive characteristics*		
Infertility diagnosis		
Male	41 (31.1)	17 (27.8)
Female	41 (31.1)	22 (36.1)
Unexplained	50 (37.9)	22 (36.1)
Protocol		
Antagonist	15 (11.4)	6 (9.8)
Flare	23 (17.4)	10 (16.4)
Luteal phase	94 (71.2)	45 (73.8)

The majority of women who were selected for the analysis but not included in the GC DNAm analysis did not have sufficient DNA extracted from their granulosa cells. Only three women were excluded because their DNAm data failed to meet quality control standards (e.g. two samples where > 5% of probes yielded detection *p*-values > 0.001, one sample that was an outlier in principal components analyses). High NO_2_ exposure was defined as > 34.1 ppb and low exposure was defined as < 11.7 ppb. High supplemental folic acid intake was defined as ≥ 800 µg/day and low intake was defined as < 400 µg/day

*BMI*, body mass index; *DNAm*, DNA methylation; *GC*, granulosa cell; *IQR*, interquartile range; *NO*_*2*_, nitrogen dioxide

### Epigenetic age acceleration

In univariate analyses, epigenetic age based on the mural GC clock (GCmAge) was moderately correlated with chronological age (r = 0.51, *p*-value = < 0.001), while epigenetic age based on the Horvath Pan-Tissue clock (DNAmAge) (r = − 0.07, *p*-value = 0.59) and the GrimAge clock (r = 0.07, *p*-value = 0.58) had little to no correlation with chronological age (Additional file 1: Figure S1). After adjusting for the surrogate variables (which were used as a proxy for heterogeneity in cellular composition and estimated using the sva package in R), the correlation between GCmAge and chronological age strengthened (r = 0.58, *p*-value = < 0.001) and DNAmAge became moderately correlated (r = 0.38, *p*-value = 0.002) and GrimAge became strongly correlated (r = 0.77, *p*-value = < 0.001, respectively) with chronological age. Age acceleration, calculated using the residual method, as estimated from the surrogate variable-adjusted clocks were moderately correlated with one another for DNAmAge acceleration and GCmAge acceleration (r = 0.49, *p*-value = < 0.001), but neither were associated with GrimAge acceleration.

We did not observe any associations between NO_2_ exposure or supplemental folic acid intake and any of the GC age acceleration variables with adjustment for age and with further adjustment for the surrogate variables or other potential confounders (i.e. BMI, education, and stimulation protocol) (Table 2). For example, in the fully adjusted model, women with high NO_2_ exposure had an adjusted mean difference of 0.52 (95% CI − 1.41, 2.45) for DNAmAge acceleration, − 0.21 (95% CI − 1.42, 1.00) for GrimAge acceleration, and − 0.57 (95% CI − 2.36, 1.22) for GCmAge acceleration compared to women with low exposure. Similarly, women with high supplemental folic acid intake had an adjusted mean difference of 1.28 (95% CI − 0.59, 3.15) for DNAmAge acceleration, 0.34 (95% CI − 0.85, 1.53) for GrimAge acceleration, and 0.40 (95% CI − 1.36, 2.16) for GCmAge acceleration compared to women with low intake.

**Table 2:** Associations between exposure to NO_2_, supplemental folic acid intake, and age acceleration of granulosa cells

	Age adjusted		Age + surrogate variables adjusted		Fully adjusted*	
	Mean difference	95% CI	Mean difference	95% CI	Mean difference	95% CI
*High versus Low NO*_*2*_* exposure*						
Horvath clock	− 1.13	− 6.10, 3.83	0.53	− 1.32, 2.38	0.52	− 1.41, 2.45
GrimAge clock	− 1.94	− 7.55, 3.68	− 0.18	− 1.36, 1.01	− 0.21	− 1.42, 1.00
GC clock	− 0.94	− 2.78, 0.91	− 0.80	− 2.51, 0.92	− 0.57	− 2.36, 1.22
*High versus low supplemental folic acid intake*						
Horvath clock	1.61	− 3.36, 6.57	1.40	− 0.43, 3.22	1.28	− 0.59, 3.15
GrimAge clock	− 1.99	− 7.62, 3.64	0.22	− 0.96, 1.41	0.34	− 0.85, 1.53
GC clock	0.18	− 1.69, 2.04	0.41	− 1.32, 2.13	0.40	− 1.36, 2.16

Age acceleration was calculated by regressing biological age (as predicted by various aging clocks) on chronological age and using the resulting residuals. Values that are positive indicate a faster aging process while negative values indicate a slower aging process. High NO_2_ exposure was defined as > 34.1 ppb and low exposure was defined as < 11.7 ppb. High supplemental folic acid intake was defined as ≥ 800 µg/day and low intake was defined as < 400 µg/day

*CI*, confidence interval; *GC*, granulosa cell; *NO*_*2*_, nitrogen dioxide

*Models were adjusted for age and the three surrogate variables as well as body mass index, education (≤ College degree, Graduate degree), and protocol (Antagonist/Flare, Luteal phase)

### Epigenome-wide association studies (EWAS of 474,545 CpGs)

**Fig. 1:**
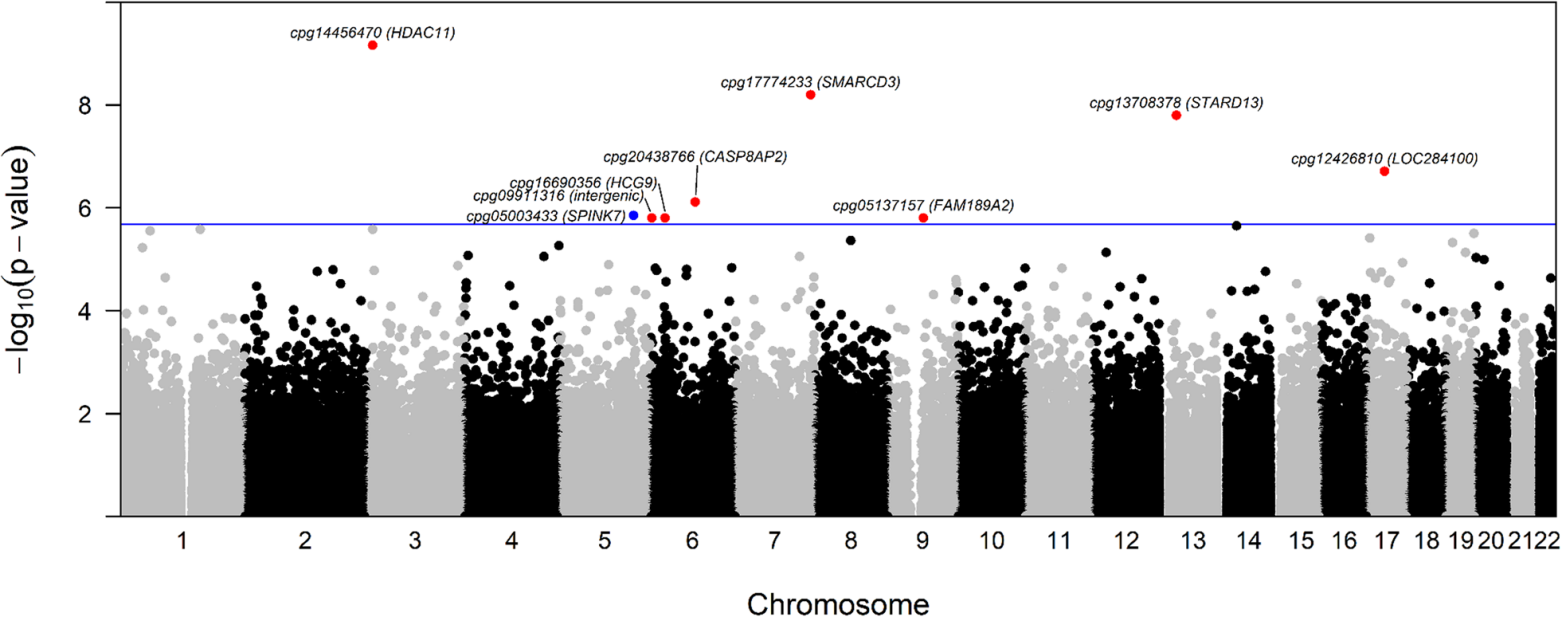
Manhattan plot of the epigenetic loci in granulosa cells that are differentially methylated with high versus low NO_2_ exposure. The x-axis shows the genomic location of the individual CpG sites. The y-axis shows the − log_10_(raw *p*-values) from models relating high NO_2_ exposure to CpG methylation, adjusting for age and three surrogate variables. The horizontal line depicts the Bonferroni adjusted *p*-value threshold. Blue dots indicate a positive association between NO_2_ exposure and DNA methylation and red dots indicate a negative association between NO_2_ exposure and DNA methylation. Significant findings after adjustment for multiple comparison were annotated with CpG ID (closest gene)

**Fig. 2:**
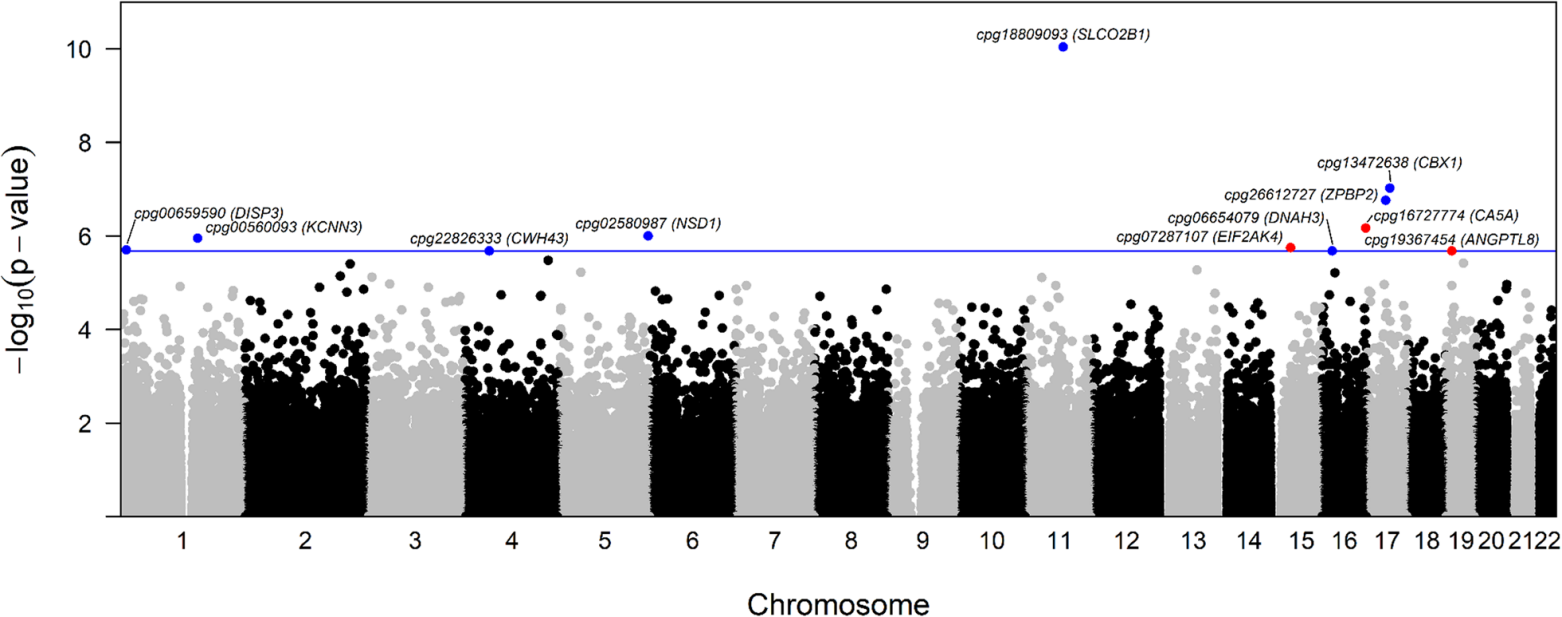
Manhattan plot of the epigenetic loci in granulosa cells that are differentially methylated with high versus low supplemental folic acid intake. The x-axis shows the genomic location of the individual CpG sites. The y-axis shows the − log_10_(raw *p*-values) from models relating high supplemental folic acid intake to CpG methylation, adjusting for age and three surrogate variables. The horizontal line depicts the Bonferroni adjusted *p*-value threshold. Blue dots indicate a positive association between supplemental folic acid intake and DNA methylation and red dots indicate a negative association between supplemental folic acid intake and DNA methylation. Significant findings after adjustment for multiple comparison were annotated with CpG ID (closest gene)

For the age and surrogate variable-adjusted EWAS of NO_2_, we identified nine differentially methylated CpGs (FDR *q*-values < 0.1), eight of which exhibited lower DNA methylation with high versus low exposure (Fig. 1, Additional file 1: Table S1). Differences in DNAm, calculated from Beta-values, ranged between − 0.071 and 0.036, with the largest magnitude of effect and smallest *p*-value at cg14456470, which is upstream of *Histone Deacetylase 11* (*HDAC11*). For the age and surrogate variable-adjusted EWAS of supplemental folic acid, we identified eleven differentially methylated CpGs (FDR *q*-values < 0.1), eight of which had higher DNAm with high versus low folic acid intake (Fig. 2, Additional file 1: Table S2). Differences in DNAm, calculated from Beta-values, ranged between − 0.038 and 0.094, with the largest magnitude of effect and smallest *p*-value at cg1880909, which is upstream of *Solute Carrier Organic Anion Transporter Family Member 2B1* (*SLCO2B1*). QQ plots and lambdas for both EWAS models indicated some but fairly modest inflation (NO_2_ lambda = 1.05, Folic acid lambda = 1.09; Additional file 1: Figure S2). Volcano plots (Additional file 1: Figure S3) show that, in general, higher NO_2_ exposure was more commonly associated with lower DNAm and higher supplemental folic acid intake was more frequently associated with higher DNAm. We also explored whether additional adjustments for other potential confounders affected our interpretations, and for both analyses, adjustment for BMI, education, and stimulation protocol resulted in similar associations.

**Fig. 3:**
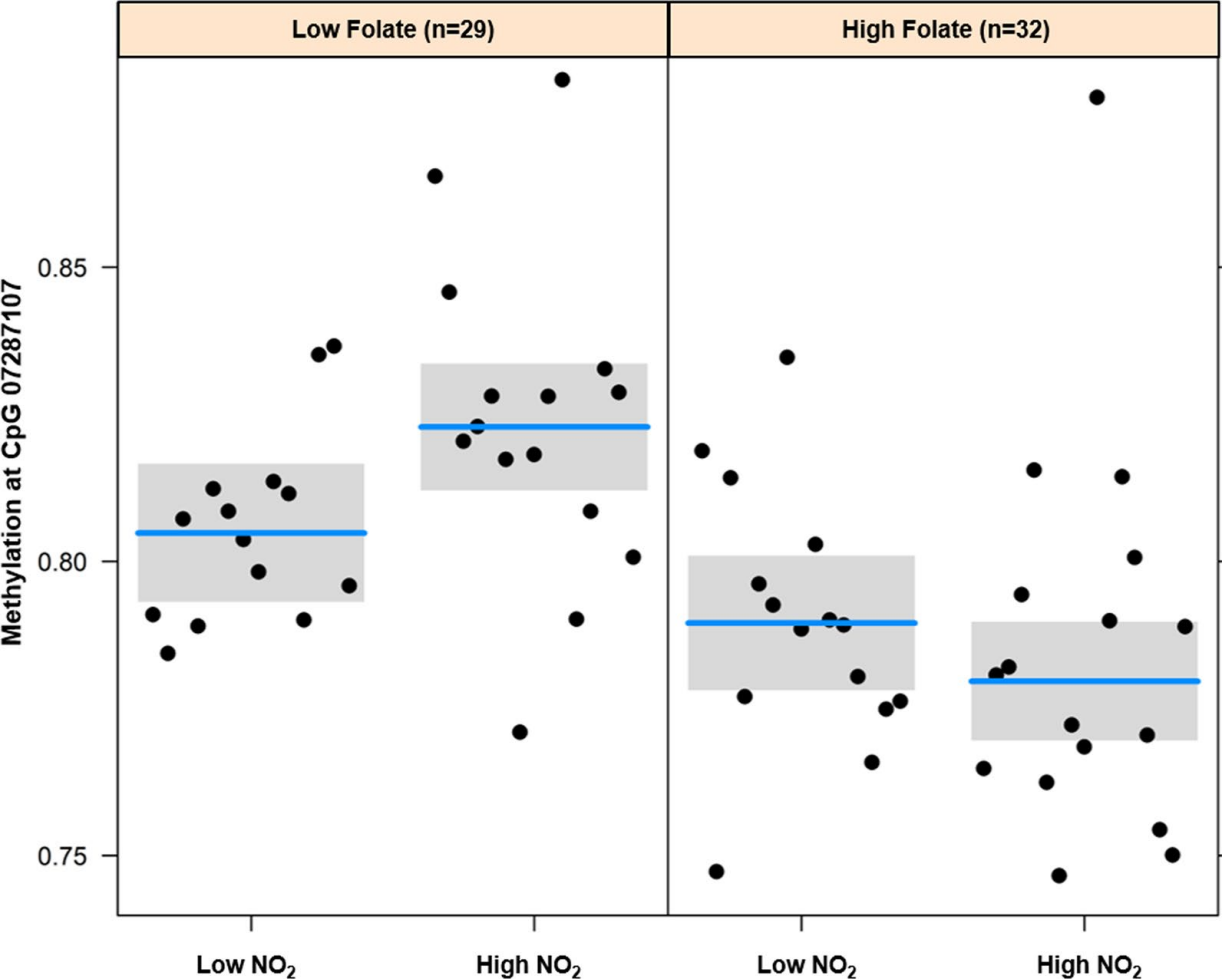
Effect modification of the association between high versus low NO_2_ exposure and methylation of cg07287107 (*EIF2AK4*) by supplemental folic acid intake. Models were adjusted for age and three surrogate variables. The *p*-value for interaction between NO_2_ and supplemental folic acid on DNAm at cg07287107 was 0.037

There was no overlap between the CpGs that were differentially methylated with high vs. low NO_2_ exposure and those associated with high vs. low supplemental folic acid intake. We then tested whether there were any statistical interactions between NO_2_ and supplemental folic acid for the 20 identified CpGs above. Only cg07287107 exhibited a statistically significant interaction after adjustment for age and three surrogate variables (interaction *p*-value = 0.037) (Fig. 3). Among women with low supplemental folic acid, those with high NO_2_ had 1.7% higher DNAm at this CpG compared to women with low NO_2_ exposure. Among women with high supplemental folic acid intake, there was no difference in DNAm associated with NO_2_ exposure. Of note, this interaction was highly influenced by the choice of covariates. Most notably, the interaction was only evident after adjustment for the third surrogate variable, which was highly correlated with methylation at this CpG site. Interestingly, cg07287107 has been identified as a methylation quantitative trait loci (mQTL), one potential explanation for this finding is that the 3rd SV is a marker of genetic ancestry [25].

### Enriched gene ontology (GO) terms and Kyoto encyclopedia of genes and genomes (KEGG) pathways

Since we only identified a small number of CpGs associated with either NO_2_ exposure or supplemental folic acid intake at an FDR threshold of 0.1, we utilized the 250 CpGs with the smallest *p*-values from each EWAS for enrichment tests. While no GO enrichment tests yielded FDR *q*-values < 0.1, seven GO terms were enriched at *p*-value < 0.001 for high vs. low NO_2_ exposure, including terms related to regulation of carbohydrate and protein metabolism, postsynaptic potential and dendrite development, as well as cell membrane components and exocytosis (Table 3). Among the CpGs associated with high supplemental folic acid, eleven GO terms were enriched at *p*-value < 0.001, including terms related to estrous cycle, learning, cognition, synaptic organization and transmission, as well as cell size and components of neuronal cell bodies. We also identified two enriched KEGG pathways (*p*-value < 0.001): hsa04810 (Regulation of actin cytoskeleton), which was enriched among the NO_2_ associated CpGs, and hsa04151 (PI3K-Akt signaling pathway), which was enriched among the supplemental folic acid associated CpGs.

**Table 3:** GO terms that were enriched at a *p*-value < 0.001 among the top 250 CpGs from each EWAS of high NO_2_ exposure and high supplemental folic acid

Exposure	GO ID	Description	*p*-value
NO_2_	GO:0006109	Regulation of carbohydrate metabolic process	2.35E−04
NO_2_	GO:0032271	Regulation of protein polymerization	4.15E−04
NO_2_	GO:2000463	Positive regulation of excitatory postsynaptic potential	6.07E−04
NO_2_	GO:0006887	Exocytosis	6.81E−04
NO_2_	GO:0016358	Dendrite development	9.46E−04
NO_2_	GO:0045121	Membrane raft	1.32E−05
NO_2_	GO:0098857	Membrane microdomain	1.36E−05
Folic acid	GO:0007611	Learning or memory	9.30E−05
Folic acid	GO:0044849	Estrous cycle	1.11E−04
Folic acid	GO:0050808	Synapse organization	1.60E−04
Folic acid	GO:0008361	Regulation of cell size	2.49E−04
Folic acid	GO:0050890	Cognition	2.81E−04
Folic acid	GO:0035584	Calcium-mediated signaling using intracellular calcium source	4.52E−04
Folic acid	GO:0090066	Regulation of anatomical structure size	5.73E−04
Folic acid	GO:0032535	Regulation of cellular component size	7.56E−04
Folic acid	GO:0050804	Modulation of chemical synaptic transmission	8.05E−04
Folic acid	GO:0099177	Regulation of trans-synaptic signaling	8.19E−04
Folic acid	GO:0043025	Neuronal cell body	1.58E−04

High NO_2_ exposure was defined as > 34.1 ppb and low exposure was defined as < 11.7 ppb. High supplemental folic acid intake was defined as ≥ 800 µg/day and low intake was defined as < 400 µg/day

*EWAS*, epigenome-wide association study; *GO*, gene ontology; *NO*_*2*_, nitrogen dioxide

## Discussion

Contrary to our initial hypothesis, in our pilot study we did not find any independent association or interaction between TRAP exposure, supplemental folic acid intake, and DNAm age acceleration of GCs. We did, however, discover 20 unique differentially methylated CpGs that were associated with either TRAP exposure or supplemental folic acid intake and multiple enriched GO terms. Our results suggest that GC DNAm could be a plausible mechanism linking environmental exposures, such as air pollution and supplemental folic acid, to ovarian function. It is also possible that that these DNA methylation differences are not involved in the causal mechanism, but instead are reflective of other processes such as altered gene expression or transcription factor binding, in which case our observations may serve as fingerprints of the exposure but may not be causal mediators.

There are several plausible reasons why we did not observe a relation between TRAP exposure and supplemental folic acid intake and DNAm age acceleration of GCs. The first, and perhaps most obvious, is that there is no relation between these exposures and ovarian aging, particularly through DNAm pathways. However, this lack of association could also be due to the poor utility of these metrics to predict ovarian aging. While some studies have found GrimAge acceleration [26] and others GCmAge acceleration [23] to be associated with ovarian reserve and outcomes of ovarian stimulation, the literature is not entirely consistent [27]. It is also possible that our etiologic time window, particularly for TRAP exposure, was incorrectly specified and that shorter or longer-term exposure might be more important. For supplemental folic acid, the woman’s MTHFR genotype could be an important effect modifier [20] and by not taking this into account this may have masked an association. Future research is needed to address many of these questions moving forward.

Consistent with previous research in blood leucocytes and placenta [13–15], we also found that, in general, higher exposure to air pollution was associated with lower methylation at specific CpG sites in GCs. TRAP exposure is known to increase the production of reactive oxygen species [28], leading to DNA damage, which then might interfere with the ability of methyltransferases to interact with DNA [29], resulting in hypomethylation of cytosine residues at CpG sites. It is also possible that these reactive oxygen species might directly alter the expression of genes belonging to DNA methylation machinery [30]. Of the nine differentially methylated CpG sites linked to NO_2_ exposure, several are located near genes that have been implicated in oogenesis. For instance, our top hit, cg14456470, is located upstream of a histone deacetylase, *HDAC11*—this gene has been shown to play a role in meiotic spindle formation, chromosome alignment and segregation, and mRNA transcription during pig and mouse oocyte maturation by regulating α-tubulin acetylation and histone modifications [31, 32]. We also identified cg20438766, which is located in the promoter region of the *CASP8AP2*. This gene is involved in a wide variety of physiological functions including the regulation of cell cycle progression, apoptotic signal transduction, transcriptional activation, and histone expression. Of most relevance to our results though, are findings from mice models showing that *CASP8AP2* is indispensable for embryogenesis, particularly at the pre-implantation stage [33].

While the majority of CpGs associated with NO_2_ exposure tended to have lower methylation with higher exposure, the majority of CpGs that were associated with folic acid exhibited the opposite relationship where higher supplemental folic acid intake tended to be associated with increased methylation. This is in line with some [20] but not all [34] previous research on this topic and congruent with our initial hypothesis given that supplemental folic acid plays a key role in the production of S-adenosyl-methionine, the universal methyl donor. There were eleven differentially methylated CpG sites associated with high vs. low supplemental folic acid intake. The top site was one located upstream of *SLCO2B1*, a gene that is expressed throughout the body, including the ovary, and encodes the 709-amino acid protein OATP2B1. This protein transports a variety of endogenous and exogenous substrates into cells, two of which are steroid precursors, dihydroepiandrosterone-3-sulfate and estrone-3-sulfate [35]. The second hit associated with supplemental folic acid intake was cg13472638, located downstream of the *CBX1*. This gene encodes a highly conserved nonhistone protein, which may play an important role in the epigenetic control of chromatin structure and gene expression. Research in mice suggests that expression of the *CBX1* is enhanced in older germinal vesicle stage oocytes [36] and may be a marker of oocyte aging. Interestingly, one of the top GO terms associated with high supplemental folic acid intake was estrous cycle, further implicating the potential importance of this nutrient in the epigenetic regulation of folliculogenesis.

Of brief note, while we found no evidence of an interaction between supplemental folic acid and TRAP exposure on DNAm age acceleration of GCs, we did identify one CpG site (cg07287107) located upstream of the *EIF2AK4* gene, in which there was a significant interaction. This gene encodes the enzyme eukaryotic translation initiation factor 2 alpha kinase 4, which phosphorylates the alpha subunit of eukaryotic translation initiation factor-2 to downregulate protein synthesis in response to varied cellular stresses [37]. This in turn can upregulate cytoprotective/DNA repair and cell-cycle machinery. Both vehicle exhaust and folic acid supplements have been shown to independently affect the DNAm profile of this gene in animal tissues [38, 39]. Transcriptome profiling of young- and middle-aged mouse ovaries has also identified eIF2 signaling as one of the top pathways that is downregulated with age, further implicating this gene in ovarian function [40]. Using the mQTL database we also found that cg07287107 is an mQTL, whose methylation levels may be influenced by multiple single nucleotide polymorphisms (SNPs) that are in cis, and have minor allele frequencies ranging between 0.10 and 0.47 [25]. Thus, it is possible that the interaction that we detected is reflective of a gene-environment interaction, driven by nearby genetic variation, but we did not have genetic data to explicitly test for this. Given our limited sample size, it is highly likely that we were underpowered to detect other CpGs where there may be an interaction between supplemental folic acid and TRAP. It is also possible that air pollution and folic acid have interactive effects at a broader biological level, impacting gene expression or similar biological pathways, but possibly not observable at level of CpG-specific methylation. Thus, larger studies and those that integrate functional genomic markers like RNA-sequencing will be needed to confirm these preliminary findings and take a more expansive analysis throughout the genome.

A somewhat surprising finding was the number of neurological GO terms including postsynaptic potential, dendrite development, learning, cognition, cell size and components of neuronal cell bodies, and synaptic organization and transmission that were associated with both TRAP exposure and supplemental folic acid. These results might provide some insight into a potential mechanism underlying the observed associations between maternal preconception air pollution and folic acid intake and risk of certain birth defects, specifically spina bifida and anencephaly, and child neurodevelopment [41, 42]. While it is generally recognized that paternal exposures can induce epigenetic alterations in sperm which can in turn affect offspring health [43], less attention has focused on the potential for maternal, preconception environmental exposures to affect offspring through the epigenome of oocytes. Although it is an intriguing hypothesis, there are a couple strong assumptions that must hold for these findings to be true. First, that the DNAm responses in GCs mirrors that of oocytes and second, that these changes in DNAm at specific CpGs would persist following the dynamic reprogramming that occurs in early embryonic development.

Our study had several limitations that should be considered when interpreting our results. First, consistent with previous studies that have measured DNAm in somatic cells of the follicular fluid, our DNA yield was low, and the reasons for this are not completely understood. Since our DNA yield was not different across exposure categories, we were less concerned about this affecting the validity of our results, but this did greatly affect our final sample size and statistical power. Thus, future work is needed to better understand how to optimize DNA collection in somatic cells of the follicular fluid to improve the efficiency of studies moving forward. Second, we did not specifically isolate GCs and therefore our samples may have contained small amounts of thecal or epithelial cells. While we adjusted for surrogate variables in an attempt to control for cellular heterogeneity or other residual confounding, this is a reference-free approach and thus there still is the possibility that the observed differences in methylation may be related to the cellular composition of follicular fluid. Third, while we used validated measures of ambient air pollution and supplemental folic acid, measurement error is still likely. This most likely resulted in bias toward the null but would have further limited our ability to differentiate signal from noise in our data. Fourth, given our small sample size (and our lack of a replication cohort), false positive findings are possible. As such, our results should be treated as hypothesis generating. Finally, given the design of our study, we were only able to include women undergoing controlled ovarian stimulation prior to IVF. If infertility patients, as compared to fertile women, have a different DNAm profiles in their GCs then the generalizability of these findings to a broader population of women may be limited. Despite these limitations, our study had several strengths including the prospective design, the use of a novel and biologically relevant biospecimen to assess DNAm signatures, high quality air pollution and dietary assessments, and rigorous statistical methods. By leveraging this highly phenotyped cohort of women undergoing IVF, we also had the unique ability to examine the influence of environmental and dietary factors on biological aging and epigenetic regulation of ovarian somatic cells, which, to date, had only been investigated in animal models.

In summary, while our study found no relation of TRAP or supplemental folic acid intake with markers of DNAm age acceleration in GCs, we did observe many differentially methylated CpGs that were associated both exposures and were located near genes linked to reproductive and fertility outcomes. This suggests that differences in GC DNAm could be a plausible mechanism underlying the effects of TRAP and supplemental folic acid on ovarian function. Our results may also have implications for certain pregnancy and offspring health outcomes as alterations in GC methylation as a result of preconception exposures could potentially affect oocyte methylation and transmit damage to the following generation. Future research to improve the assessment of epigenetic signatures within and across reproductive cells is warranted.

## Materials and methods

### Study population

To address our research questions, we leveraged existing data from the Environment and Reproductive Health (EARTH) Study, a prospective cohort designed to evaluate environmental and dietary determinants of fertility [44]. In brief, all women 18–45 years old presenting for infertility evaluation at the Massachusetts General Hospital Fertility Center (2004–2019) were eligible for the EARTH study. Upon enrollment, all women completed detailed baseline questionnaires, had anthropometric measurements taken, and provided a spot urine and blood sample. Women were then followed through their infertility treatments until discontinuation or live birth.

For this pilot project, eligible women were those who had undergone controlled ovarian stimulation with oocyte retrieval between 2006 and 2016 (the timeframe when air pollution and diet data were available) and had a stored follicular fluid sample available for analysis. To ensure we had the widest possible exposure contrasts for both TRAP and supplemental folic acid intake, we selected 33 women from each of the following groups: high TRAP/low supplemental folic acid, low TRAP/low supplemental folic acid, high TRAP/high supplemental folic acid, and low TRAP/high supplemental folic acid as defined below. Because we anticipated follicular fluid may have a low DNA extraction yield, we selected 33 samples in each group with the goal of obtaining sufficient DNA in at least 24 samples so that the final sample would include 96 women.

### Air pollution and diet assessment

Women provided their residential address at study entry, which were geocoded using ArcGIS, and linked to a validated, nationwide spatio-temporal model of nitrogen dioxide (NO_2_), a marker of TRAP, at a 1 km^2^ resolution. These daily NO_2_ concentrations were estimated by a model which uses satellite remote sensing data in combination with land use regression [45]. We averaged the daily NO_2_ concentrations over the 3 months prior to the start of controlled ovarian stimulation as this roughly corresponds to the proposed window of follicular development [46]. High and low exposure to TRAP was defined as an average NO_2_ exposure > 75th percentile (34.1 ppb) and < 25th percentile (11.7 ppb), respectively. These cutoffs were chosen to optimize exposure contrast. As such, we did not have any women with average NO_2_ exposures between 11.7 and 34.1 ppb. Diet was assessed with a validated food frequency questionnaire in which women reported how often they consumed 131-item food items and supplements during the previous year. Folate intake with this questionnaire has been validated against diet records (r = 0.77) [47] and plasma (r = 0.54) [48] folate concentrations, with high validity and reproducibility. High supplemental folic acid intake was defined as consuming ≥ 800 µg/day while low intake as defined as < 400 µg/day. We focused primarily on supplemental folic acid and the 800 µg/day cutoff because a previous analysis from the EARTH Study cohort showed that high supplemental folic acid intake (e.g. ≥ 800 µg/day) (and to a lesser extent total folate intake) attenuated the adverse association between preconception TRAP exposure and lower probability of live birth following IVF. Similar to the rationale for NO_2_, to optimize exposure contrast, we did not have any women with supplemental folic acid intakes 400–799 µg/day.

### Follicular fluid collection

Women underwent oocyte retrieval following 9–14 days of controlled ovarian stimulation. During this procedure, a follicular fluid sample was taken from women’s first three follicles with a 16 G needle. Each sample was collected in a separate tube prepared with 1 ml of flushing media. Once the oocytes were removed, the follicular fluid was centrifuged to separate the supernatant and pellet and resulting aliquots were stored at − 80 °C. The pellet is presumed to be mostly GCs although there may have been a small proportion of epithelial or thecal cells, as we did not perform a purification step for GCs. The stored GCs from the first aspirated follicle was shipped to the Emory Integrated Genomics Core on dry ice, blinded to exposure.

### Quality control and processing of DNA methylation data

DNA extraction was performed with the QIAamp UCP DNA Micro Kit (Qiagen, Hilden, Germany), quantified using the Quant-iT dsDNA broad range assay kit (ThermoFisher, Waltham, MA), and assessed for quality on a 2% agarose gel. Preparation of DNA for the array was performed according to the Illumina Infinium HD Assay Methylation Protocol Guide. The samples were randomly distributed across the well plate, to reduce the potential for batch effects. The Emory University Integrated Genomics Core performed bisulfite modification using the EZ DNA Methylation Kit (Zymo Research, Irvine, CA), and measured DNAm throughout the genome with the Illumina MethylationEPIC Beadarray (Illumina, San Diego, CA) following the manufacturer’s protocol. Functional normalization and beta-mixture quantile (BMIQ) normalization were performed to reduce technical artefacts. After excluding probes on the X chromosome, those that are cross hybridizing, those with SNPs at the target CpG or within one base pair of the target CpG, and those with low variability (standard deviation of beta-values < 0.02), 474,545 probes were available for analysis.

### Accounting for unmeasured confounding

We also recognize that accounting for differences in cellular heterogeneity or technical artefacts is a critical component of studies that utilize DNA methylation microarray data. There is no current reference methylome for follicular fluid samples, thus we could not directly estimate and adjust for the cellular composition of our samples. Instead, we utilized surrogate variable analysis (*sva* package in R) to estimate the major sources of variation in our data and identified three surrogate variables. These may be reflective of heterogeneity in cellular composition or other unmeasured confounding and were included as covariates in our models.

### Estimation of epigenetic age

We estimated epigenetic age using three different clocks. First, we calculated the Horvath Pan-tissue clock (DNAmAge), which was developed to predict chronological age from more than 8000 samples where DNA was obtained from 51 healthy tissues [49]. This clock estimates age based on a weighted sum of 353 CpG sites that were selected using an elastic net regression. Second, we calculated the GrimAge clock which was developed with the goal of predicting lifespan. This clock, which consists of 1030 CpG sites, was built using a two-step method that took plasma protein levels, smoking, sex and chronological age into account [50]. The primary justification for deriving the GrimAge epigenetic clock was that it had the strongest correlation with chronological age, ovarian reserve, and outcomes of ovarian stimulation in a previous analysis of GC DNAm clocks [26]. Third, we estimated epigenetic age via the GC clock (GCmAge), which was developed to estimate chronological age based on a weighted sum of 296 CpG sites—this clock was trained on 27 samples of mural GCs [23] which were combined with 621 blood and epithelial cell samples that were used to train the Horvath Skin & Blood clock [51]. To calculate age acceleration, we regressed DNAmAge, GrimAge, and GCmAge on reported age (calculated as retrieval date minus birthdate) and extracted the residuals.

### Statistical analyses

We used Spearman’s correlations to quantify the association between reported and predicted age values based on the three epigenetic clocks. Multivariable linear regression models were used to evaluate the association of TRAP exposure and supplemental folic acid intake with age acceleration of the follicular fluid adjusted for age, BMI (continuous, kg/m^2^), education (less than or equal to college vs. graduate school), and protocol (luteal vs. flare or antagonist), and surrogate variables. TRAP exposure and supplemental folic acid intake were modelled as binary variables (high vs. low). The beta-coefficients from this model that are positive indicate a faster aging process while negative values indicate a slower aging process. To evaluate DNAm at individual CpG sites, we used robust linear regression adjusted for age and three surrogate variables after applying an unsupervised dimension reduction by excluding CpGs with low variability (standard deviation < 0.02) to reduce multiple testing burden [52]. We corrected for multiple testing by estimating the false discovery rate (FDR) and considered findings with a *q* < 0.1 to be notable. Differences in methylation were derived from Beta-values. To provide biological context to the sets of CpGs that may be impacted by NO_2_ exposure and supplemental folic acid intake, we used clusterProfiler to perform enrichment analyses [53]. Overrepresentation for Gene Ontology (GO) terms and Kyoto Encyclopedia of Genes and Genomes (KEGG) pathways were determined using the enrichGO and enrichKEGG functions, which perform one-sided Fisher’s Exact tests. RStudio was used for all analyses (Version 4.0; R Foundation for Statistical Computing, Vienna, AT).

## Supplementary Information

**Additional file 1**. **Supplemental Figure 1.** Scatterplots depicting the association between chronological age and predicted epigenetic age of the granulosa cells according to the Horvath Pan-tissue, Grim Age, and Granulosa Cell clocks. **Supplemental Figure 2.** QQ plots of EWAS results from the analysis of NO_2_ (A) and supplemental folate (B), where models were adjusted for age and for three surrogate variables. **Supplemental Figure 3.** Volcano Plots of EWAS results from the analysis of NO_2_ (A) and supplemental folate (B), where models were adjusted for age and for three surrogate variables; CpGs that yielded FDR q-values < 0.1 are highlighted with red outlines. **Supplemental Table 1.** Differentially methylated CpGs associated with high versus low NO2 exposure that yielded FDR q-values < 0.10. **Supplemental Table 2.** Differentially methylated CpGs associated with high versus low supplemental folate intake that yielded FDR q-values < 0.10.

## Data Availability

The datasets used and analyzed during the current study are available from the corresponding author on reasonable request.

## Abbreviations

DNAm: DNA methylation
EWAS: Epigenome-wide association study
FDR: False discovery rate
GC: Granulosa cells
GO: Gene ontology
KEGG: Kyoto encyclopedia of genes and genomes
NO_2_: Nitrogen dioxide
TRAP: Traffic-related air pollution
