# Supplementary material for: Traffic-related air pollution and supplemental folic acid intake in relation to DNA methylation in granulosa cells
Source: Clin Epigenetics. 2023 May 13;15:84. doi: 10.1186/s13148-023-01503-y (PMC10183139; doi:10.1186/s13148-023-01503-y)
Supplement: Supplementary file 1 — Additional file 1. Supplemental Figure 1. Scatterplots depicting the association between chronological age and predicted epigenetic age of the granulosa cells according to the Horvath Pan-tissue, Grim Age, and Granulosa Cell clocks. Supplemental Figure 2. QQ plots of EWAS results from the analysis of NO2 (A) and supplemental folate (B), where models were adjusted for age and for three surrogate variables. Supplemental Figure 3. Volcano Plots of EWAS results from the analysis of NO2 (A) and supplemental folate (B), where models were adjusted for age and for three surrogate variables; CpGs that yielded FDR q-values < 0.1 are highlighted with red outlines. Supplemental Table 1. Differentially methylated CpGs associated with high versus low NO2 exposure that yielded FDR q-values < 0.10. Supplemental Table 2. Differentially methylated CpGs associated with high versus low supplemental folate intake that yielded FDR q-values < 0.10. [file 13148_2023_1503_MOESM1_ESM.docx]

**Supplemental Figure 1**. Scatterplots depicting the association between chronological age and predicted epigenetic age of the granulosa cells according to the Horvath Pan-tissue, Grim Age, and Granulosa Cell clocks.


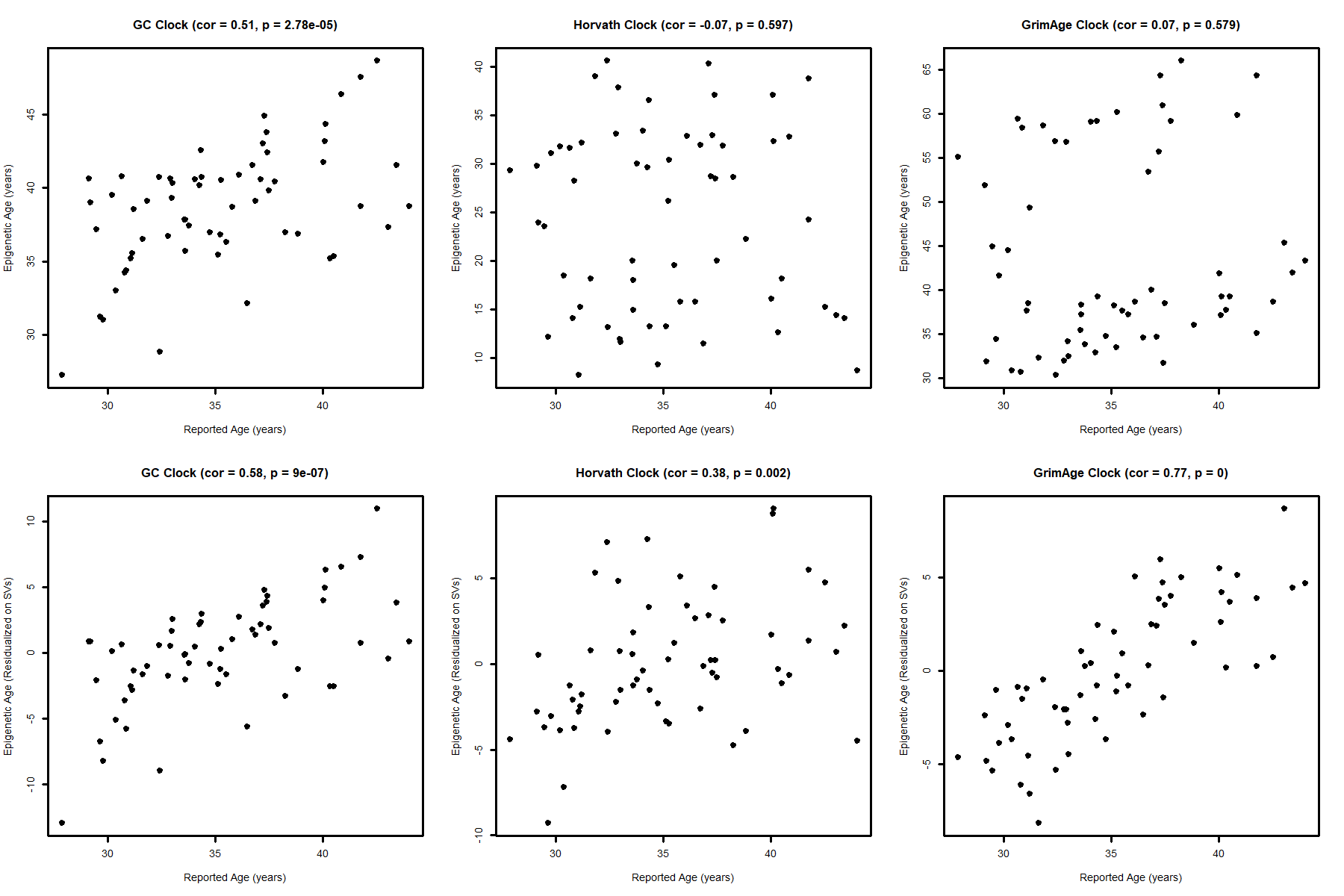


**Supplemental Figure 2:** QQ plots of EWAS results from the analysis of NO_2_ (A) and supplemental folate (B), where models were adjusted for age and for three surrogate variables.


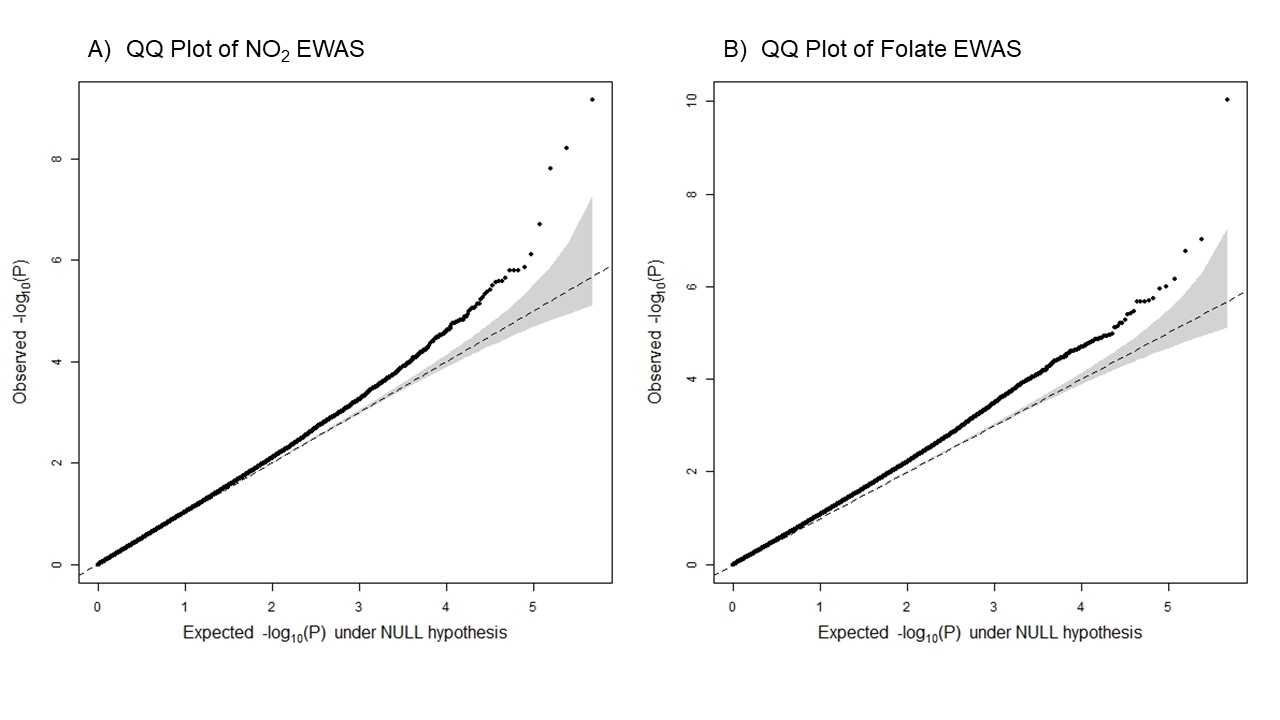


**Supplemental Figure 3:** Volcano Plots of EWAS results from the analysis of NO_2_ (A) and supplemental folate (B), where models were adjusted for age and for three surrogate variables; CpGs that yielded FDR q-values < 0.1 are highlighted with red outlines.


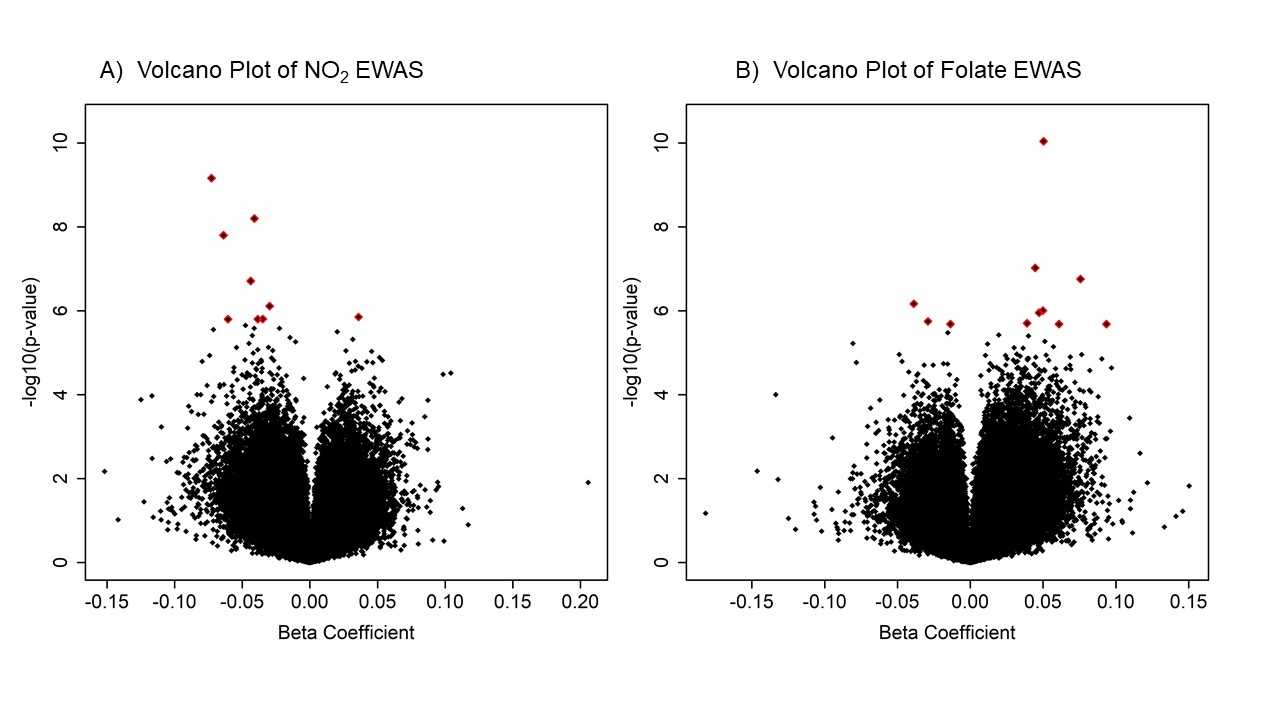


**Supplemental Table 1**. Differentially methylated CpGs associated with high versus low NO_2_ exposure that yielded FDR q-values < 0.10.

|  | **Age & SV adjusted** | | **Multivariable adjusted*** | |  |  |  |
| --- | --- | --- | --- | --- | --- | --- | --- |
| **CpG ID** | **β** | **P-value** | **β** | **P-value** | **Chromosome** | **Closest Gene** | **Region^†^** |
| cg14456470 | -0.073 | 6.91E-10 | -0.071 | 8.11E-10 | chr3 | *HDAC11* | Upstream |
| cg17774233 | -0.041 | 6.34E-09 | -0.035 | 3.13E-05 | chr7 | *SMARCD3* | Upstream |
| cg13708378 | -0.064 | 1.59E-08 | -0.058 | 5.39E-06 | chr13 | *STARD13* | 5'UTR |
| cg12426810 | -0.044 | 1.95E-07 | -0.045 | 1.47E-08 | chr17 | *LOC284100* | Downstream |
| cg20438766 | -0.030 | 7.76E-07 | -0.032 | 5.32E-08 | chr6 | *CASP8AP2* | TSS1500 |
| cg05003433 | 0.036 | 1.40E-06 | 0.035 | 5.71E-07 | chr5 | *SPINK7* | Body |
| cg09911316 | -0.035 | 1.58E-06 | -0.033 | 5.32E-07 | chr6 | intergenic | - |
| cg05137157 | -0.038 | 1.58E-06 | -0.040 | 4.03E-06 | chr9 | *FAM189A2* | TSS1500 |
| cg16690356 | -0.061 | 1.59E-06 | -0.059 | 5.85E-06 | chr6 | *HCG9* | Body |

Abbreviations: DNAm, DNA methylation; FDR, false discovery rate; NO2, nitrogen dioxide; SV, surrogate variable. High NO_2_ exposure was defined as >34.1 ppb and low exposure was defined as <11.7 ppb. The β is the estimated differential DNAm associated with high vs. low NO_2_ exposure.

*****Model was additionally adjusted for BMI, education (≤College degree, Graduate degree), and protocol (Antagonist/Flare, Luteal phase).

**^†^** Annotations were confirmed in UCSC Genome Browser (hg19); CpGs without Illumina annotations were annotated as *upstream* or *downstream* if the CpG was within 100kb of a gene in UCSC.

**Supplemental Table 2**: Differentially methylated CpGs associated with high versus low supplemental folate intake that yielded FDR q-values < 0.10.

|  | **Age & SV adjusted** | | **Multivariable adjusted*** | |  |  |  |
| --- | --- | --- | --- | --- | --- | --- | --- |
| **CpG ID** | **β** | **P-value** | **β** | **P-value** | **Chromosome** | **CpG ID** | **Region^†^** |
| cg18809093 | 0.050 | 9.19E-11 | 0.052 | 1.68E-09 | chr11 | *SLCO2B1* | Upstream |
| cg13472638 | 0.045 | 9.54E-08 | 0.046 | 3.90E-07 | chr17 | *CBX1* | Downstream |
| cg26612727 | 0.076 | 1.76E-07 | 0.072 | 1.80E-06 | chr17 | *ZPBP2* | 1stExon |
| cg16727774 | -0.039 | 6.83E-07 | -0.039 | 1.08E-05 | chr16 | *CA5A* | Body |
| cg02580987 | 0.050 | 9.92E-07 | 0.048 | 0.000213 | chr5 | *NSD1* | Body |
| cg00560093 | 0.047 | 1.12E-06 | 0.046 | 6.57E-07 | chr1 | *KCNN3* | ExonBnd |
| cg07287107 | -0.029 | 1.79E-06 | -0.027 | 4.08E-05 | chr15 | *EIF2AK4* | Upstream |
| cg00659590 | 0.039 | 1.99E-06 | 0.042 | 1.87E-07 | chr1 | *DISP3^€^* | 5'UTR |
| cg22826333 | 0.061 | 2.07E-06 | 0.064 | 2.13E-06 | chr4 | *CWH43* | TSS1500 |
| cg06654079 | 0.094 | 2.08E-06 | 0.098 | 4.94E-05 | chr16 | *DNAH3* | Body |
| cg19367454 | -0.014 | 2.09E-06 | -0.013 | 6.20E-08 | chr19 | *ANGPTL8^€^* | TSS1500 |

Abbreviations: DNAm, DNA methylation; FDR, false discovery rate; SV, surrogate variable. High supplemental folate intake was defined as ≥800 µg/day and low intake was defined as <400 µg/day. The β is the estimated differential DNAm associated with high vs. low supplemental folate intake.

*****Model was additionally adjusted for BMI, education (≤College degree, Graduate degree), and protocol (Antagonist/Flare, Luteal phase).

**^†^**Annotations were confirmed in UCSC Genome Browser (hg19); CpGs without Illumina annotations were annotated as upstream or downstream if the CpG was within 100kb of a gene in UCSC.

*^€^*Gene name IDs were updated via UCSC genome browser (*PTCHD2* to *DISP3*, and *C19orf80* to *ANGPTL8*).
